# Fibroblast-like synovial cells from normal and inflamed knee joints differently affect the expression of pain-related receptors in sensory neurones: a co-culture study

**DOI:** 10.1186/ar2112

**Published:** 2007-01-25

**Authors:** Gisela Segond von Banchet, Jonny Richter, Marion Hückel, Christina Rose, Rolf Bräuer, Hans-Georg Schaible

**Affiliations:** 1 Institute of Physiology, University of Jena, Teichgraben 8, D-07740 Jena, Germany; 2 Current address: Roche Diagnostics GmbH, D-82377 Penzberg, Germany; 3 Institute of Pathology, University of Jena, Ziegelmühlenweg, D-07740 Jena, Germany

## Abstract

Innervation of the joint with thinly myelinated and unmyelinated sensory nerve fibres is crucial for the occurrence of joint pain. During inflammation in the joint, sensory fibres show changes in the expression of receptors that are important for the activation and sensitization of the neurones and the generation of joint pain. We recently reported that both neurokinin 1 receptors and bradykinin 2 receptors are upregulated in dorsal root ganglion (DRG) neurones (the cell bodies of sensory fibres) in the course of acute and chronic antigen-induced arthritis in the rat. In this study, we begin to address mechanisms of the interaction between fibroblast-like synovial (FLS) cells and sensory neurones by establishing a co-culture system of FLS cells and DRG neurones. The proportion of DRG neurones expressing neurokinin 1 receptor-like immunoreactivity was not altered in the co-culture with FLS cells from normal joints but was significantly upregulated using FLS cells from knee joints of rats with antigen-induced arthritis. The proportion of DRG neurones expressing bradykinin 2 receptors was slightly upregulated in the presence of FLS cells from normal joints but upregulation was more pronounced in DRG neurones co-cultured with FLS cells from acutely inflamed joints. In addition, the expression of the transient receptor potential V1 (TRPV1) receptor, which is involved in inflammation-evoked thermal hyperalgesia, was mainly upregulated by co-culturing DRG neurones with FLS cells from chronically inflamed joints. Upregulation of neurokinin 1 receptors but not of bradykinin 2 and TRPV1 receptors was also observed when only the supernatant of FLS cells from acutely inflamed joint was added to DRG neurones. Addition of indomethacin to co-cultures inhibited the effect of FLS cells from acutely inflamed joints on neurokinin 1 receptor expression, suggesting an important role for prostaglandins. Collectively, these data show that FLS cells are able to induce an upregulation of pain-related receptors in sensory neurones and, thus, they could contribute to the generation of joint pain. Importantly, the influence of FLS cells on DRG neurones is dependent on their state of activity, and soluble factors as well as direct cellular contacts are crucial for their interaction with neurones.

## Introduction

The inflammatory response in organs is produced by numerous inflammatory cell types. These cell types communicate with each other in order to develop an appropriate inflammatory reaction. A large amount of information on the mechanisms of interaction of different inflammatory cells has been obtained from co-culture systems of different cell types, such as T cells and monocytes [1-3], T cells and endothelial cells [4], T cells and fibroblasts [5-7], monocytes and fibroblasts [8,9], and macrophages and fibroblasts [10-12]. These data have established the importance of both cell-cell contacts and mediators for the production of the inflammatory activity.

Most tissues are innervated, and nerve fibres play an important role in inflammatory diseases. The activation of nociceptive sensory afferent fibres ('pain fibres') evokes pain, a major symptom of inflammatory diseases [13]. Furthermore, there is growing evidence that primary afferent neurones as well as sympathetic nerve fibres influence the inflammatory process through efferent processes [14-16]. Despite the functional cross-talk between the inflammatory process and neurones, the mechanistic analysis of interactions between non-neuronal inflammatory cells and neurones has not been carried out in great detail. Recently, a first report appeared on the influence of neurones in the central nervous system on T cells and the potential role of neurone-T cell interactions on experimental autoimmune encephalomyelitis [17].

The somata of primary afferent neurones are located in the dorsal root ganglia (DRG). Similar to the sensory endings, the somata of these neurones express ion channels and receptors that are important for the activation and/or sensitization of these neurones, and they thus seem to represent the total primary afferent neuron in this respect [18]. In addition, the expression of ion channels and receptors in the somata is altered during peripheral inflammation. We recently took DRGs from normal rats and from rats with unilateral acute and chronic antigen-induced arthritis (AIA) in the knee, cultured them for one day and determined which proportion of DRG neurones express receptors for bradykinin (BK) and substance P (neurokinin 1 (NK1) receptors). In lumbar DRGs but not in cervical DRGs from AIA rats we found a pronounced increase in the proportion of neurones exhibiting BK receptors and NK1 receptors [19]. The upregulation of these receptors found in this study and in other studies on inflammation [20,21] is thought to be involved in the inflammatory pain response because both BK and substance P activate and/or sensitize proportions of primary afferent neurones for mechanical stimuli, which is a mechanism of mechanical hyperalgesia [22-26]. Indeed AIA rats show limping of the inflamed knee and a lowered pain threshold when pressure is applied to the knee [19]. In addition, the transient receptor potential V1 (TRPV1) receptor is an ion channel that is involved in thermal inflammatory hyperalgesia [27-29]. Some studies [30-33], but not others [34,35], have identified an upregulation of TRPV1 receptors in DRGs in inflammatory models.

We have begun to identify mechanisms that cause the upregulation of BK, NK1 and TRPV1 receptors in DRG neurones. In the present study we co-cultured DRG neurones with fibroblast-like synovial (FLS) cells from either normal knee joints or from acutely or chronically inflamed knee joints from AIA rats. FLS cells are key players in the propagation of joint inflammation and joint destruction during rheumatoid arthritis [36-39] whereas DRG neurones are key players in the development of chronic pain [18]. We addressed three questions. First, is the expression of these receptors in DRG neurones influenced by the presence of FLS cells? Second, do FLS cells from normal and inflamed knee joints exert different effects on receptor expression in DRG neurones? Third, are effects of FLS cells on DRG neurones mediated by soluble mediators (is the supernatant of FLS cells sufficient?) or is the presence of the FLS cells with cellular contacts important? Preliminary results have been reported [40,41].

## Materials and methods

### Induction of joint inflammation

In 17 10-week-old female Lewis rats (Charles River, Sulzfeld, Germany), an inflammation was induced in the right knee joint. In the first step the rats received a subcutaneous injection of 500 μg antigen (methylated BSA; Sigma, Deisenhofen, Germany) in 500 μl saline emulsified with 500 μl complete Freund's adjuvant (Sigma; supplemented to 2 mg/ml heat-killed *Mycobacterium tuberculosis *strain H37RA, Difco, Detroit, MI, USA). In addition, an intraperitoneal injection of 2 × 10^9 ^heat-inactivated *Bordetella pertussis *(Chiron Behring, Marburg, Germany) was performed on the same day. The same immunisation procedure was repeated 7 days later. After a further 14 days, a sterile solution of antigen (methylated BSA), 500 μg in 50 μl saline, was injected into the right knee joint cavity (day 0). Either 3 days (acute AIA) or 20 to 28 days (chronic AIA) after induction of inflammation in the knee joint, the rats were killed by cervical dislocation during ether anaesthesia. In total, 17 untreated rats of the same age and sex were used as normal control animals. All rats were used for the preparation of FLS cells. All procedures complied with the regulations of the Thuringian Commission for Animal Protection.

### Preparation of fibroblast-like synovial cells

Explant cultures of FLS cells were prepared from the knee joints of normal rats or from rats 3 days (acute phase) or 20 to 28 days (chronic phase of inflammation) after induction of AIA. The patella and the menisci of the joints with adjacent synovial tissue were separated and cultured in 24-well plates in DMEM (Gibco, BRL, Eggenstein-Leopoldshafen, Germany) containing 20% fetal calf serum (FCS, [Gibco]), 0.1 mg/ml streptomycin (Grünenthal, Aachen, Germany), 100 U/ml penicillin (Jenapharm, Jena, Germany), 2 mmol/l glutamine (Gibco), 10 mM Hepes (Gibco), and 1 mmol/l sodium pyruvate (Gibco) for 7 days at 37°C in a humidified incubator gassed with 5% CO_2 _in air. During this time, out-growing FLS cells emerged from the tissue. In the first 7 days the medium was replaced daily. After 7 days the residual tissue was removed and 2 days later the cells were transferred to new plates. For this purpose the cells were washed with PBS and incubated for 2 to 4 minutes in PBS containing 0.25% trypsin and 0.02% EDTA (Gibco). Thereafter, the cells were collected, washed with DMEM containing 20% FCS and disseminated. After another 3 to 6 days the cells were transferred into new plates. For the co-culture, cells were used after the third to fifth passage. FLS cells were slowly cooled down with isopropanol to -70°C in DMEM containing 10% dimethylsulfoxide and stored at -192°C over liquid nitrogen until co-culturing.

### Primary culture of dorsal root ganglion neurones

Normal male Wistar rats, 60 days old, were sacrificed with a lethal dose of ether. DRGs from all segments of the spinal cord were dissected. Ganglia were incubated at 37°C with 215 U/ml collagenase type II (Paesel and Lorei, Hanau, Germany) dissolved in Ham's F12 medium (Gibco) for 100 minutes. After washing with Ca^2+^- and Mg^2+^-free PBS, the ganglia were placed in DMEM (Gibco) containing 10,000 U/ml trypsin (Sigma) for 11 minutes at 37°C. The cells were dispersed by gentle agitation and aspiration with a fire polished Pasteur-pipette. The dispersed cells were collected by centrifugation (500 × g, 5 minutes), washed 3 times in DMEM and centrifuged. The cell pellets were suspended in Ham's F-12 medium containing 10% heat-inactivated horse serum (Gibco), 100 U/ml penicillin (Gibco), 100 μg/ml streptomycin (Gibco), and 100 ng/ml nerve growth factor (NGF; Paesel and Lorei). On average, 200 to 300 DRG neurones were plated on poly-L-lysine- (200 μg/ml) coated glass cover slips (diameter 13 mm) situated in 35/10 mm dishes and maintained for 1 night at 37°C in a humidified incubator gassed with 3.5% CO_2 _and air. After this overnight setting period, the co-cultures of DRG neurones and the FLS cells were prepared.

### Co-culture of FLS cells and DRG neurones

Two days before co-culturing, the FLS cells (from normal rats or from rats with acute or chronic AIA) were thawed and cultured in DMEM containing 20% FCS in a concentration of 2 × 100 cells/well in 24-well dishes. After 2 days, the FLS cells were incubated with 0.25% trypsin (Gibco) and 0.02% EDTA (Sigma) in DMEM for 2 to 4 minutes at 37°C. Thereafter, the cells were washed 3 times with DMEM (containing 100 ng/ml NGF) and added to the glass cover slips with the cultured DRG neurones (see above) in a concentration of 10^5^/ml. The two cell types were co-cultured for 24 hours in DMEM containing 100 ng/ml NGF maintained at 37°C in a humidified incubator gassed with 5% CO_2 _in air. As a control, either only DRG neurones or only FLS cells were cultured and handled in the same way as the co-cultures. After culturing for 24 hours, all cells on the glass cover slips were fixed and used for immunocytochemical labelling of the NK1, the BK 2 (B2) and the TRPV1 receptors.

In addition, in some experiments, DRG neurones were cultured in medium containing only the supernatant of FLS cells. These FLS cells were isolated from either normal knee joints or knee joints at the acute (day 3) or chonic state (20 to 28 days) of AIA and then cultured for 2 days. The supernatants of these FLS cells were added to the DRG neurones, which were kept for 24 hours at 37°C in a humidified incubator gassed with 5% CO_2 _in air, and, in addition, 100 ng/ml NGF were administered.

Furthermore, co-cultures of DRG neurones and FLS cells from normal or acutely and chronically inflamed knee joints were made in a medium containing either the cyclooxygenase (COX) inhibitor indomethacin (1 μmol/l; Calbiochem, Bad Soden/Ts, Germany), an antibody against IL-6 (1 μmol/l; BioTrend, Köln, Germany) or IgG from normal rat (1 μmol/l; BioTrend). These co-cultures were kept at 37°C in a humidified incubator gassed with 5% CO_2 _in air for 24 hours in DMEM containing 100 ng/ml NGF.

### Detection of bradykinin 2 receptors

Because a reliable B2 receptor antibody was not available, we used BK-gold conjugates for labelling of B2 receptors. The BK-gold conjugates were prepared as described earlier [42]. In brief, 1 μmol BK (Bachem, Heidelberg, Germany) was dissolved in 500 μl HEPES (20 mmol/l, pH 7.5). This solution was added to 6 nmol sulfo-N-hydroxy-succinimido Nanogold reagent (BioTrend), dissolved in 500 μl ddH_2_O, and incubated for 1 hour at room temperature. To separate BK-gold conjugates from unbound BK, a membrane centrifugation (Amicon microcon-10 system) was used. The BK-gold conjugate was dissolved in PBS containing 0.1% BSA (Sigma), 0.2 mol/l sucrose (Sigma), 4 μg/ml leupeptin (Sigma) and 10 mmol/l sodium azide (Sigma). This solution was aliquoted and stored at -20°C for a maximum of three months.

The cells were fixed with 2% paraformaldehyde in 0.1 mol phosphate buffer (pH 7.2) for 30 minutes. After washing with PBS (20 mmol/l, pH 7.4), the cells were pre-treated with 50 mmol/l glycine in PBS and, thereafter, with 5% BSA and 0.1% gelatine in PBS for 30 minutes. Then the cells were washed with 0.1% acetylated BSA (BSA-C; BioTrend) and incubated overnight with 0.3 nmol/ml BK-gold in PBS containing 0.1% BSA-C, bacitracin (40 μg/ml; Sigma), leupeptin (4 μg/ml; Sigma) and chymostatin (2 μg/ml; Sigma) at 4°C in a moist chamber. Following washing with PBS plus 0.1% BSA-C and, thereafter, with PBS to remove unbound BK-gold, cells were postfixed with 2% glutaraldehyde in PBS for 10 minutes. After extensive washing with PBS and ddH_2_O, the gold particles were intensified with silver enhancer (R-Gent, pH 5.5; BioTrend) for 15 minutes at 22°C. The reaction was stopped by washing in ddH_2_O. To examine whether the binding was related to B2 receptors, 3 nmol/ml BK-gold was incubated in parallel control dishes in the presence of 1 μmol/ml D-Arg [Hyp^3^-Thi^5,8^-D-Phe^7^]-BK (Sigma), a BK analogue that specifically binds to the B2 receptor. This analogue produces a complete displacement of BK-gold.

### Immunocytochemical labelling of NK1 and TRPV1 receptors

The cover slips were transferred to 2% paraformaldehyde in 0.1 mol/l phosphate buffer (pH 7.2) plus 0.3% Triton X-100 (TX-100) for 30 minutes. After washing with PBS plus 0.3% Triton X-100 (PBS TX-100), cells were incubated with 50 mmol/l glycine in PBS TX-100 and, thereafter, with 5% BSA and 0.1% gelatine in PBS TX-100 for 30 minutes. Then the cells were washed with PBS TX-100 and incubated for 30 minutes in PBS TX-100 containing 2% normal goat serum (BioTrend). Thereafter, the cells were washed with PBS TX-100 containing 0.1% BSA-C and incubated overnight with an anti-NK1 antibody diluted 1:100 in PBS TX-100 plus 1% normal goat serum (the antibody was raised in rabbit against amino acids 393 to 407 of the rat NK1 receptor; Sigma) or with an antibody to the TRPV1 receptor (VR11-A, 1:75; Alpha Diagnostics, San Antonio, USA) at 4°C in a moist chamber. The cover slips were extensively rinsed in PBS TX-100 plus 0.1% BSA-C and, thereafter, in PBS TX-100. After washing, the cells were incubated for 4 hours at 20°C with a gold-labelled (10 nm) anti-rabbit antibody developed in goat (BioTrend), diluted 1:100 in PBS TX-100 plus 1% normal goat serum. After washing with PBS TX-100, PBS and ddH_2_O, the gold particles were intensified with silver enhancer (R-Gent, pH 5.5) for 20 minutes at 21°C. The reaction was stopped by washing in ddH_2_O. To test for unspecificity of the detection system, cells were incubated only with the secondary antibody. In these cultures no cells were labelled.

### Double-labelling with an antibody against neurone-specific enolase

To identify the neurones in the cultures, double-staining with an anti-neurone-specific enolase (NSE) antibody was used in all experiments in which B2, NK1, and TRPV1 receptor labelling was performed. After washing with PBS, the cover-slips were incubated overnight at 4°C with the anti-NSE-antibody (Sigma) developed in rabbit (diluted 1:100). Then the cover-slips were incubated with a goat anti-rabbit antibody labelled with Cy3 (diluted 1:200; Jackson ImmunoResearch, Cambridgeshire, UK) for 2 hours. After washing with PBS, all cover slips were embedded in Vectashield (Vector, Burlingame, England). In control experiments in which the primary antibody (anti-NSE) was omitted, no fluorescence signal was detectable.

### Prostaglandin E_2 _and IL-6 measurement in the supernatant of the cultures

The supernatants of cultured DRG neurones, cultured FLS cells and co-cultures of DRG neurones and FLS cells (from normal and acutely or chronically inflamed joints) were analysed for the production of prostaglandin E_2 _(PGE_2_) and IL-6. The samples were stored at -20°C until analysis. For each substance, four independent cultures were used. All samples were measured twice. The supernatants were analysed with commercial ELISA kits for PGE_2 _(Cayman Chemicals, Ann Arbor, Michigan, USA) and for rat IL-6 (OptEIA; BD Biosciences, Heidelberg, Germany).

### Data analysis

From each cover slip, 100 structurally intact and NSE-labelled neurones were examined with a light microscope (Axioplan 2, Zeiss, Germany) coupled to a CCD video camera and an image analysing system (KS 300, Zeiss, Germany). The mean area and mean grey value were determined for each neuronal soma. To take into account differences in the basal grey values on each coated cover slip, a relative grey value of each neurone was calculated by dividing the mean grey value of the neurone by the grey value of the cover slip background. For an unbiased discrimination of cells with or without positive labelling with the antibodies against NK1 and TRPV1 or BK-gold, neurones were considered positive if their relative grey value was above that of neurones from the control incubations, which were not treated with the antibodies against NK1 or TRPV1 receptors. In experiments with BK-gold labelling, neurones were considered positive if their relative grey value was above that of neurones from the displacement control incubations with a BK analogue that specifically binds to the B2 receptor. This value was 0.16; thus, all neurones with grey density >0.16 were considered as positive for the antibodies or B2 receptor BK-gold binding. Proportions of labelled neurones are expressed as the mean ± standard deviation. For statistical analysis we used χ^2^-tests taking into account multiple comparisons. Significant differences were acknowledged if *p *< 0.05.

## Results

### Co-culture system

**Figure 1:**
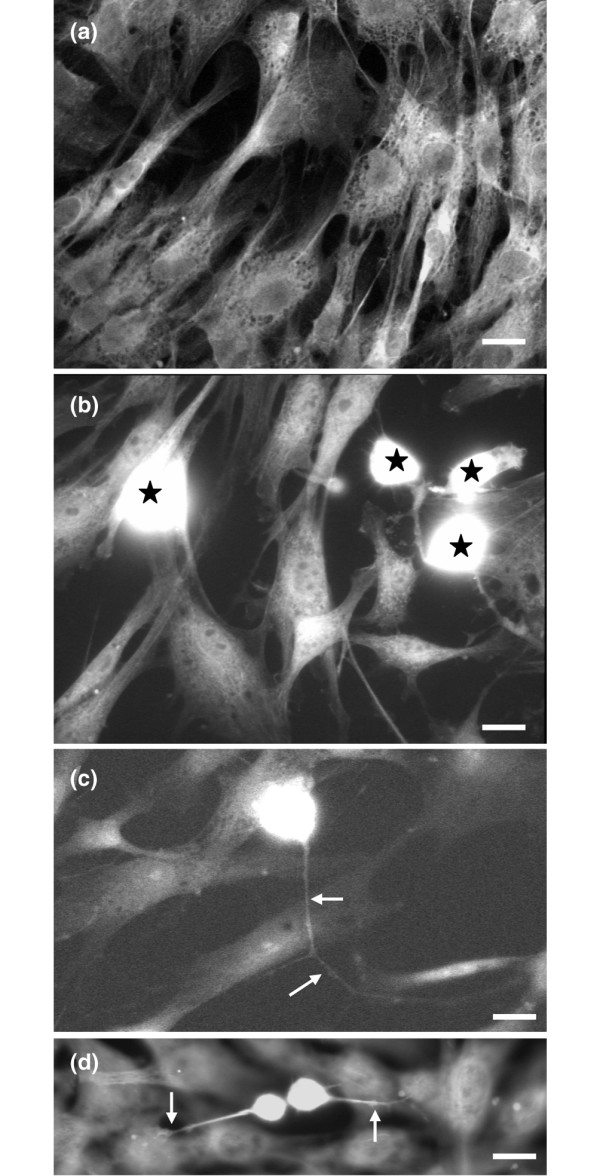
Morphology of cultured fibroblast-like synovial (FLS) cells and co-cultured dorsal root ganglion (DRG) neurones. **(a) **Mono-culture of FLS cells. **(b) **Co-culture of FLS cells and DRG neurones. The DRG neurones show neurone-specific enolase-like immunoreactivity (white staining indicated by stars). **(c,d) **Co-culture of FLS cells and DRG neurones. The processes of DRG neurones are indicated with arrows. Note the contact between the DRG neurones and the FLS cells (d). Scale bars: 10 μm (a); 30 μm (b,c); 10 μm (d).

As a first step we characterized the morphology of FLS cells and DRG neurones in the co-culture system. The FLS cells formed a flat layer composed mainly of triangular NSE-negative cells (Figure 1a). After the setting period, DRG neurones that showed strong NSE-like immunoreactivity (IR) were dispersed as single cells or small cell clusters on the FLS cell layer (Figure 1b, neurones are labelled with stars). Only very few FLS cells (maximum of 1%) showed some NSE-like IR and, therefore, the antibody against NSE is a good tool to differentiate between neurones and FLS cells. The neurones had round perikarya of varying sizes and thin neurites spanning over and along FLS cells (Figure 1b–d).

**Figure 2:**
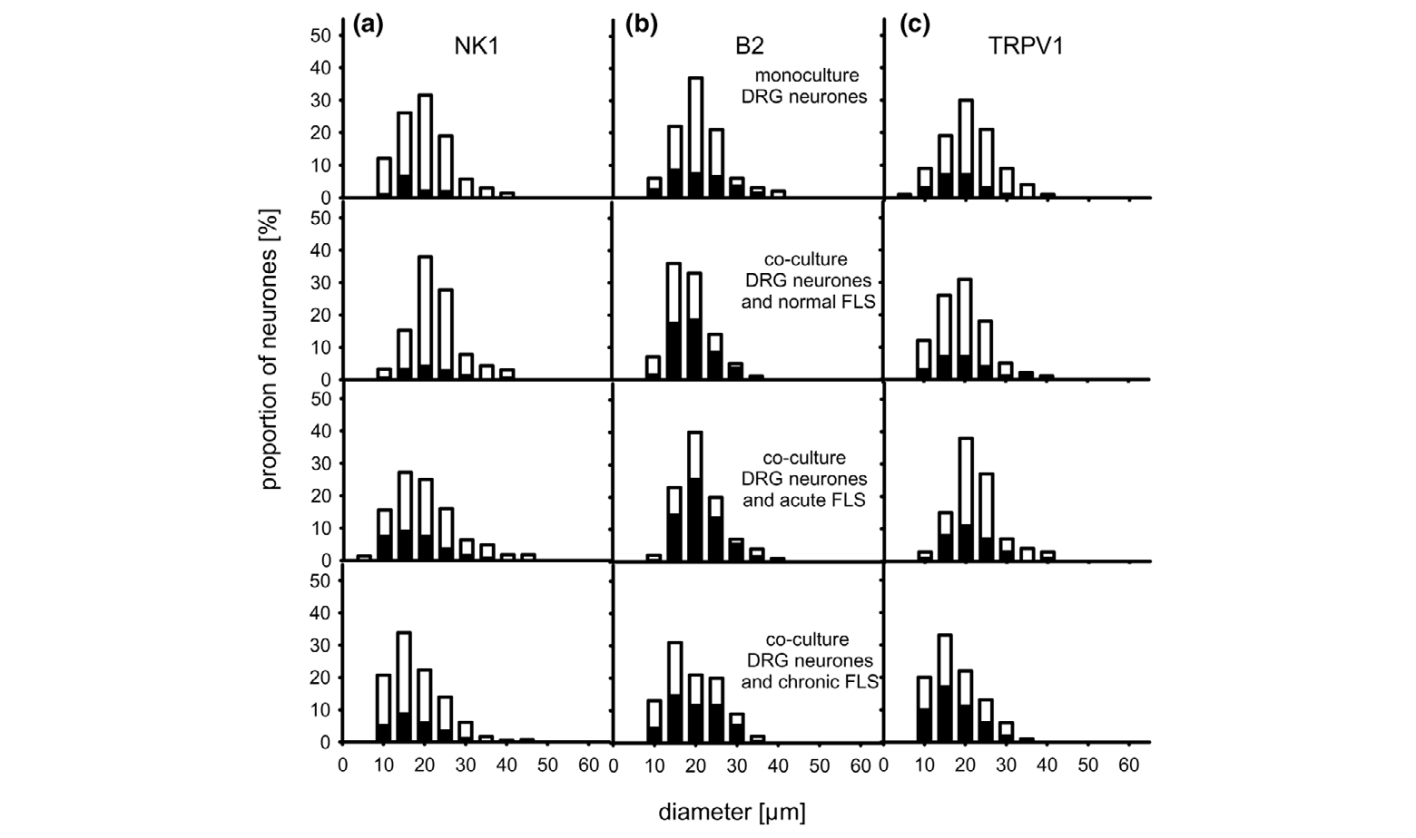
Size distribution of dorsal root ganglion (DRG) neurones under different culture conditions. The open bars in the graphs show the proportions of neurones (%) in the indicated size ranges; the black insets show the proportions of neurones that express neurokinin 1 (NK1) receptor-like immunoreactivity **(a)**, bradykinin 2 (B2) receptor labelling **(b)**, and transient receptor potential V1 (TRPV1) receptor-like immunoreactivity **(c)**. Cells were grown in DRG mono-culture (top) or together with fibroblast-like synovial (FLS) cells from normal, acutely inflamed and chronically inflamed knee joints.

DRG neurones are characterized by the size of their cell body and exhibit a typical size distribution with most cells in the small- and medium-sized range (which give rise to unmyelinated and thin myelinated axons) and fewer cells in the large-sized range (which give rise to thick myelinated axons). To compare the size distribution of DRG neurones in different cultures, we determined the diameter of neurones in the DRG mono-culture and in the co-cultures with FLS cells from acutely and chronically inflamed knee joints. The size distribution of neurones was similar under all culture conditions, indicating that the DRG cell samples were comparable (Figure 2). The morphology of FLS cells was also similar in mono-cultures and in co-cultures with DRG neurones.

### Expression of NK1, B2, and TRPV1 receptor-like IR in DRG neurones after co-culture with FLS cells

As a first approach we tested whether the presence of FLS cells from normal, acutely or chronically inflamed knee joints influences receptor expression in DRG neurones taken from normal rats. DRG neurones were fixed 24 hours after co-culturing and receptor expression was determined in NSE-labelled DRG neurones.

#### Neurokinin 1 receptor-like immunoreactivity

**Figure 3:**
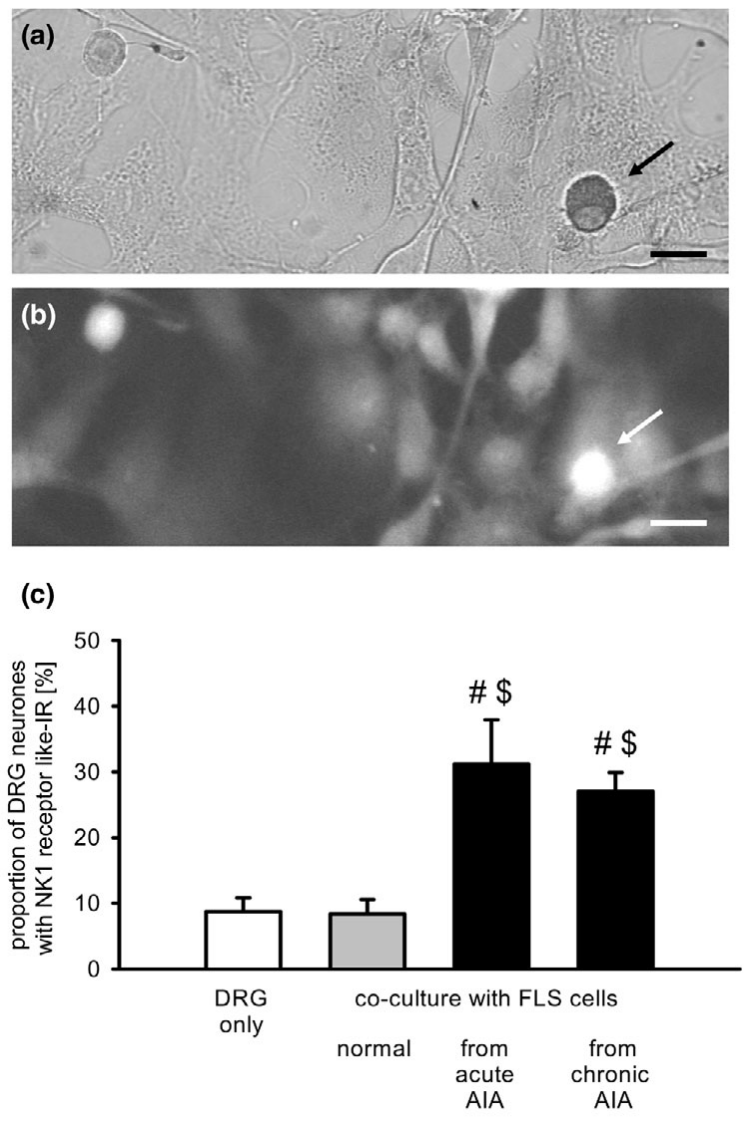
Influence of fibroblast-like synovial (FLS) cells on the expression of neurokinin 1 (NK1) receptor-like immunoreactivity (IR) in dorsal root ganglion (DRG) neurones. **(a) **Co-culture of DRG neurones from normal rats and FLS cells isolated from inflamed knee joints three days after induction of antigen-induced arthritis (AIA). The dark cell (see arrow) shows labelling for NK1 receptor-like immunoreactivity. **(b) **Same cells as in (a) double-labelled with an anti-neurone-specific enolase antibody. Scale bar, 10 μm. **(c) **Influence of FLS cells from normal and acutely and chronically inflamed knee joints on the expression of NK1 receptor-like immunoreactivity in DRG neurones from normal rats. ^#^Significant difference to DRG mono-cultures. ^$^Significant difference to co-culture with FLS cells from normal knee joints. AIA, antigen-induced arthritis.

In the standard mono-culture of DRG neurones from adult normal rats, only a small proportion of neurones showed NK1 receptor-like IR, similar to previously reported studies [19,43]. On average, 8.8 ± 2.0% of the DRG neurones (4 cultures) were labelled with the anti-NK1 receptor antibody (Figure 3c, first bar). When DRG neurones were co-cultured with FLS cells isolated from the knee joint of normal adult rats, a similar proportion of DRG neurones (8.4 ± 2.1%, 5 cultures) showed NK1 receptor-like IR (Figure 3c, second bar). However, in co-cultures of DRG neurones from normal rats and FLS cells from the inflamed knee of AIA rats, the proportion of neurones expressing NK1 receptor-like IR was significantly higher (Figure 3c, third and fourth bar). Figure 3a,b displays a cover slip with a co-culture of DRG neurones and FLS cells isolated from acutely inflamed knee joints (3 days of AIA). The dark cell in Figure 3a (see arrow) is labelled for NK1 receptor-like IR, and Figure 3b shows that this cell is also NSE-positive. Counting of double-labelled neurones showed that 31.3 ± 6.7% of the DRG neurones expressed NK1 receptor-like IR after co-culture with FLS cells isolated from knee joints of rats at 3 days of AIA (4 cultures), whereas 27.0 ± 2.9% of the DRG neurones were labelled with the anti-NK1 receptor antibody after co-culture with FLS cells isolated from the knee joint of rats at 21 to 28 days of AIA (chronic AIA, 4 cultures). At both time points, NK1 receptor expression was increased significantly compared to the mono-culture of DRG neurones and the DRG co-culture with FLS cells from normal knee joints. Black columns in Figure 2a show the proportions of neurones exhibiting NK1 receptor-like IR. Thus, FLS cells isolated from both acutely and chronically inflamed knee joints induced an upregulation of the NK1 receptor in DRG neurones from normal rats whereas a co-culture with FLS cells from normal knee joints did not. FLS cells themselves did not show NK1 receptor-like IR.

#### Bradykinin 2 receptor-like labelling

**Figure 4:**
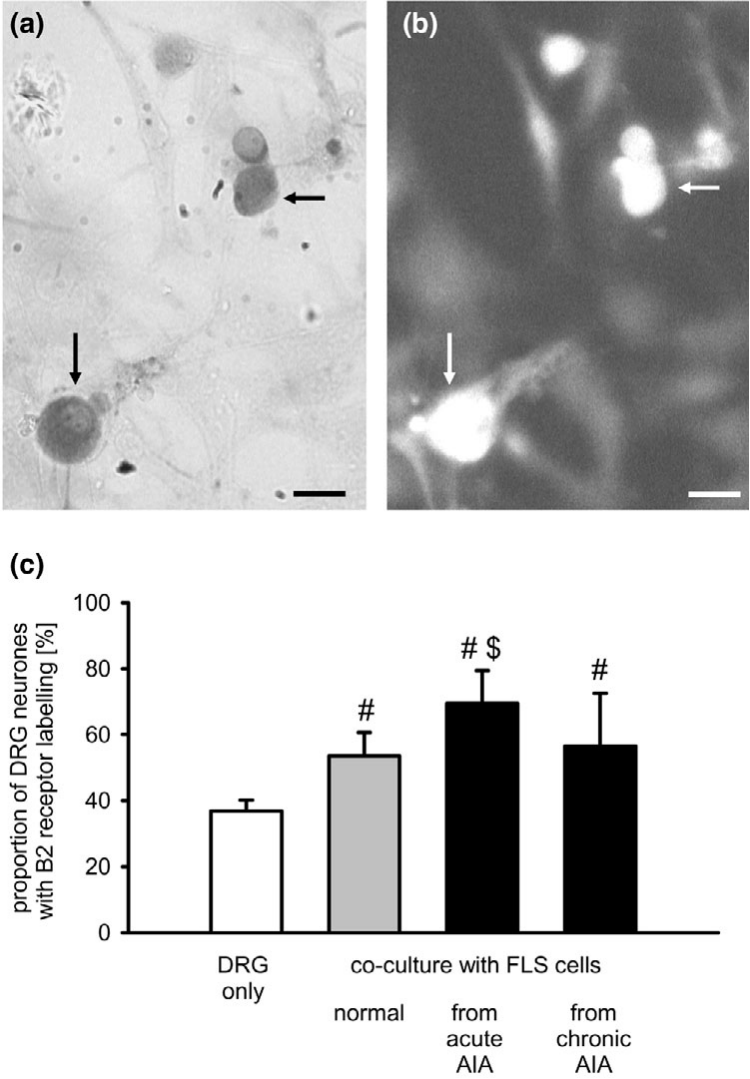
Influence of fibroblast-like synovial (FLS) cells on bradykinin 2 (B2) receptor-like labelling in dorsal root ganglion (DRG) neurones. **(a) **Co-culture of DRG neurones and FLS cells isolated after induction of acute antigen-induced arthritis (AIA). Dark DRG neurones (arrows) show bradykinin-gold labelling. **(b) **Same cells as in (a) double-labelled with an anti-neurone-specific enolase antibody. Scale bar, 10 μm. **(c) **Influence of FLS cells isolated from normal, acutely or chronically inflamed knees on B2 receptor-like labelling in DRG neurones. ^#^Significant difference to DRG mono-cultures. ^$^Significant difference to co-culture with FLS cells from normal knee joints. AIA, antigen-induced arthritis.

Because a BK analogue that specifically binds to the B2 receptor completely displaced the BK-gold particle (see Materials and methods), we conclude that BK-gold binds only to B2 receptors in this experimental approach. In the standard mono-culture of DRG neurones from normal adult rats, 36.8 ± 3.4% of all DRG neurones (5 cultures) showed B2 receptor-like labelling (Figure 4c, first bar), which is in the same range as in previous studies [19,42,44,45]. When DRG neurones and FLS cells isolated from knee joints of normal adult rats were co-cultured, on average, 53.5 ± 7.1% of the DRG neurones (4 cultures) were labelled for B2 receptors. Thus, in contrast with NK1 receptor expression, FLS cells from normal knee joints caused a significant up-regulation of the proportion of DRG neurones exhibiting B2 receptor-like labelling (Figure 4c, second bar). Compared to co-cultures of DRG neurones and FLS cells from normal joints, a significantly higher proportion of DRG neurones (on average, 69.5 ± 9.9% of the DRG neurones, 4 cultures) showed B2 receptor expression when FLS cells from acutely inflamed knee joints were used for co-culture (Figure 4c, third bar). In co-cultures of DRG neurones and FLS cells from chronically inflamed joints, 56.5 ± 16% of the DRG neurones were labelled for B2 receptors (8 cultures, not significantly different from co-cultures of DRG neurones and FLS cells from normal knee joints). Figure 4a,b displays a cover slip with a co-culture of DRG neurones and FLS cells isolated from knee joint from rats at the acute AIA stage. The size distribution of labelled neurones is shown in Figure 2b. FLS cells did not exhibit B2 receptor labelling.

#### TRPV1 receptor-like immunoreactivity

**Figure 5:**
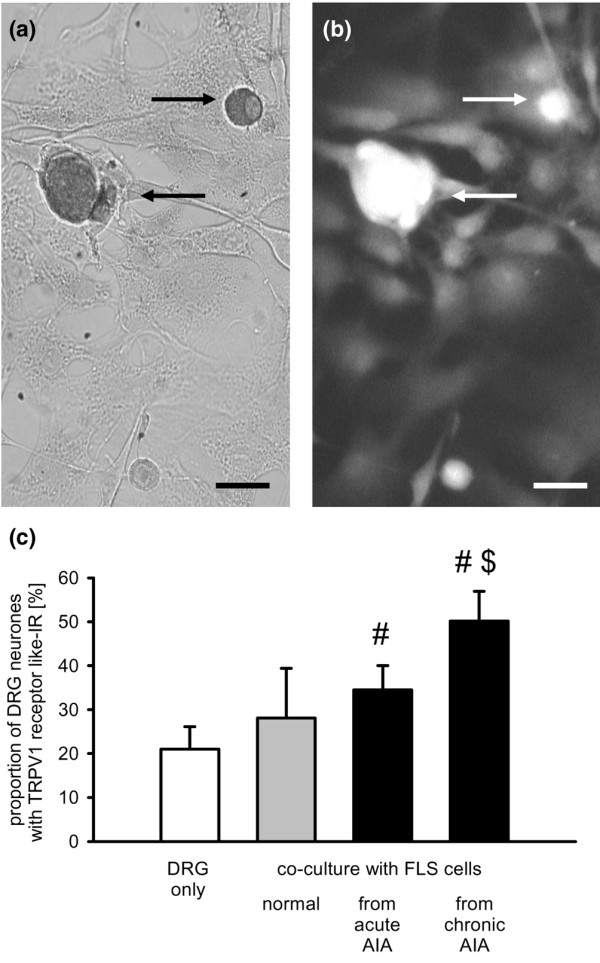
Influence of fibroblast-like synovial (FLS) cells on the expression of transient receptor potential V1 (TRPV1) receptor-like immunoreactivity in dorsal root ganglion (DRG) neurones. **(a) **Labelled DRG neurones (see arrows) expressing TRPV1 receptor-like immunoreactivity after co-culture with FLS cells isolated from acutely inflamed knee joints. **(b) **Same cells as in (a) double-labelled with an anti-neurone-specific enolase antibody. Scale bar, 10 μm. **(c) **Influence of FLS cells isolated from normal, acutely and chronically inflamed knee joints on the expression of TRPV1 receptor-like immunoreactivity in co-cultured DRG neurones. ^#^Significant difference to DRG mono-cultures. ^$^Significant difference to co-culture with FLS cells from normal knee joints. AIA, antigen-induced arthritis.

In 13 DRG mono-cultures from normal adult rats, 21.0 ± 5.1% of the neurones showed TRPV1 receptor-like IR (Figure 5c, first bar). In 7 co-cultures of DRG neurones and FLS cells isolated from normal knee joints, on average, 28.1 ± 11.3% of the DRG neurones showed TRPV1 receptor-like IR (Figure 5c, second bar), and this proportion was not different from that in DRG neurones in mono-cultures. Co-cultures of DRG neurones with FLS cells from knee joints of AIA rats yielded higher proportions of neurones exhibiting TRPV1 receptor-like IR. After co-culture with FLS cells from acutely inflamed knee joints, 34.5 ± 5.5% of DRG neurones were immunopositive for the TRPV1 receptor (4 cultures), and after co-culture with FLS cells from chronically inflamed knee joints, 50.1 ± 6.8% of the DRG neurones were immunopositive (7 cultures; Figure 5c, third and fourth bar). These proportions of immunopositive neurones were significantly higher than the proportion of those in the DRG mono-culture but only the latter value (co-cultures with FLS cells from chronically inflamed joints) was significantly higher than the proportion of immunopositive neurones in the co-culture with FLS cells from normal joints. Figure 5a,b displays a cover slip with a co-culture of DRG neurones and FLS cells isolated from acutely inflamed knee joints. Figure 2c shows the size distribution of labelled neurones. FLS cells did not show TRPV1 receptor-like IR.

### Influence of soluble FLS cell mediators on the expression of NK1, B2, and TRPV1 receptors

**Figure 6:**
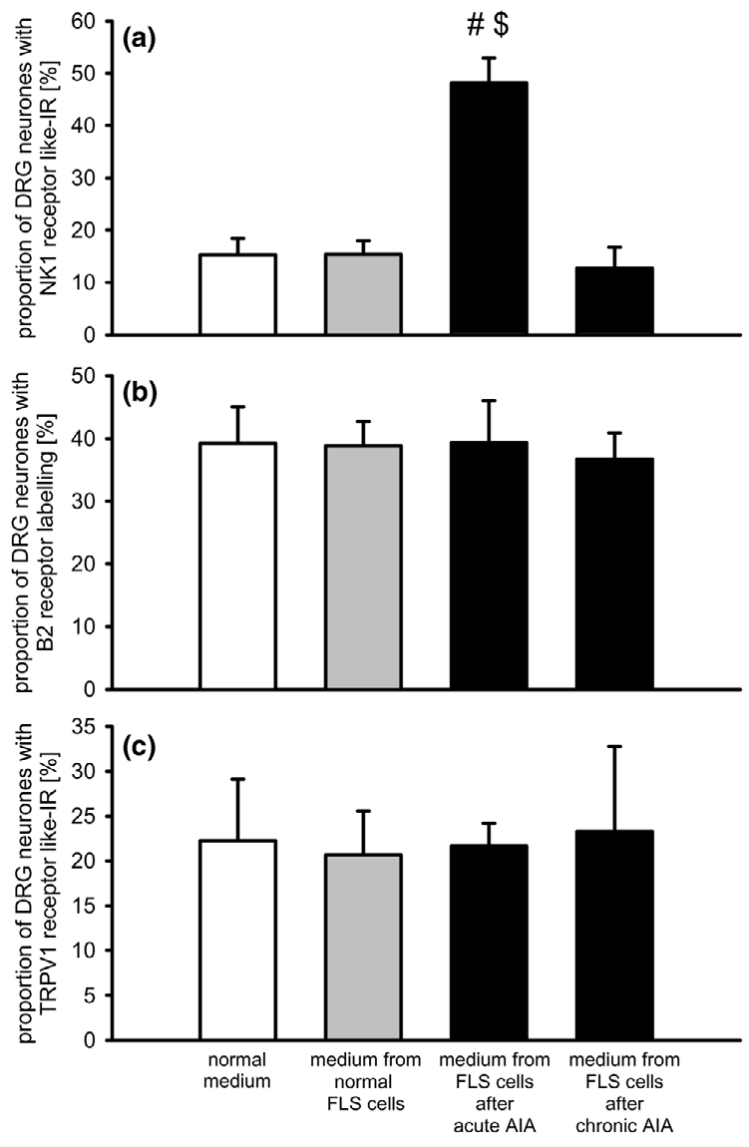
Influence of soluble mediators on the expression of neurokinin 1 (NK1), bradykinin 2 (B2), and transient receptor potential V1 (TRPV1) receptors in dorsal root ganglion (DRG) neurones. Proportion of DRG neurones with **(a) **NK1 receptor-like immunoreactivity (IR), **(b) **B2 receptor-like labelling, and **(c) **TRPV1 receptor-like immunoreactivity after culturing either in normal medium, or after administration of the supernatant of fibroblast-like synovial (FLS) cells from normal, acutely or chronically inflamed knee joints. ^#^Significant difference to DRG mono-cultures. ^$^Significant difference to co-culture with FLS cells from normal knee joints. AIA, antigen-induced arthritis.

In a second approach, we tested whether soluble mediators are responsible for the up-regulation of NK1, B2, and TRPV1 receptors that is observed in co-culture with FLS cells. We cultured DRG neurones and added only the supernatant from cultured FLS cells sampled from normal and inflamed knee joints. The results are displayed in Figure 6.

Only NK1 receptor-like IR was changed by addition of supernatant and this effect was dependent on the source of the supernatant (Figure 6a). The supernatant from FLS cells from normal knee joints did not change the proportion of neurones showing NK1 receptor-like IR. In 4 cultures, 15.4 ± 2.6% of the neurones were immunopositive, versus 15.3 ± 2.6% of the neurones in 5 control cultures with normal medium. After application of the supernatant of FLS cells from acute AIA joints, the proportion of DRG neurones with NK1 receptor-like IR significantly increased to 48.2 ± 4.7% (4 cultures). Such an effect was not seen when supernatant from FLS cells from chronically inflamed knee joints was added to the neurones. In this case, only 12.0 ± 4.0% (3 cultures) of the neurones showed NK1 receptor-like IR. By contrast, none of the supernatants influenced the proportion of DRG neurones showing B2 receptor labelling (Figure 6b, each value is from three to five cultures) and TRPV1 receptor labelling (Figure 6c, each value is from three cultures).

Because we previously found that the expression of NK1 receptor-like IR in DRG neurones is upregulated by long-term addition of either PGE_2 _[43] or IL-6 [46] to the culture medium, we measured the concentration of PGE_2 _and IL-6 in the supernatants from different cultures, and we tested whether interference of PGE_2 _and IL-6 would reduce the effect of FLS cells on NK1 receptor expression in DRG neurones. While PGE_2 _and IL-6 were below the detection level in the supernatants from DRG mono-cultures, the supernatants of FLS cells from normal joints contained both PGE_2 _and IL-6 (Table 1). The concentrations of both mediators were higher in supernatants of FLS cells from acutely inflamed joints. The concentration of PGE_2 _tended to be even higher in supernatants of FLS cells plus DRG neurones. The PGE_2 _concentration was also measured in the supernatant from chronically inflamed joints. It was even more elevated than in the supernatant of FLS cells from acutely inflamed joints but was not further enhanced by addition of DRG neurones (Table 1). IL-6 was not found to be elevated in the chronic stage of AIA [47] and was, therefore, not determined in the present study.

**Figure 7:**
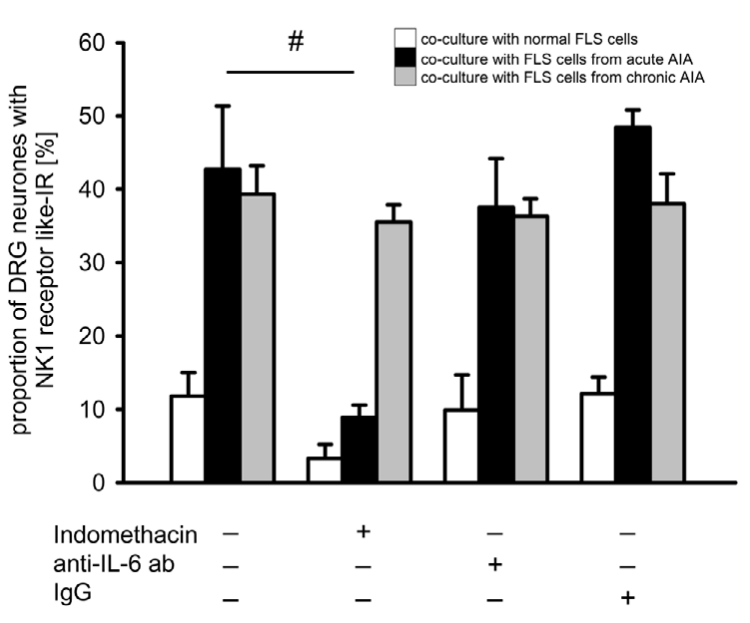
Influence of cyclooxygenase (COX) inhibition on the expression of the neurokinin 1 (NK1) receptors in dorsal root ganglion (DRG) neurones in different co-cultures with fibroblast-like synovial (FLS) cells. Proportions of DRG neurones with NK1 receptor-like immunoreactivity (IR) after culturing in normal medium, in medium with the COX inhibitor indomethacin (1 μmol/l), in medium containing an anti-IL6 antibody (1 μmol/l) or in medium containing normal rat IgG (1 μmol/l). White bars show co-cultures of DRG neurones with FLS cells from normal knee joints, black bars show co-cultures of DRG neurones with FLS cells from acutely inflamed knee joints (day 3), and grey bars show co-cultures of DRG neurones with FLS cells from chronically inflamed joints. ^#^Significant difference between untreated and indomethacin-treated co-cultures of DRG neurones and FLS cells from acutely inflamed knee joints.

The results of different treatments to interfere with PGE_2 _and IL-6 are shown in Figure 7. The expression of NK1 receptor-like IR was analysed 24 hours after co-culturing. In co-cultures of DRG neurones and FLS cells from acutely and chronically inflamed knee joints without additional treatment, we found the typical upregulation of NK1 receptor-like IR (first three columns, 4 cultures each). This was different when the COX inhibitor indomethacin (1 μmol/l) was added to the medium (columns 4 to 6). After addition of indomethacin, only few DRG neurones co-cultured with FLS cells from normal joints showed NK1 receptor-like IR, and the upregulation in the presence of FLS cells from acutely inflamed knees was prevented because, under these conditions, only 8.9 ± 1.7% of the DRG neurones showed NK1 receptor-like IR (black column). This proportion is significantly lower than the proportion of NK1 receptor-immunopositive neurones from co-cultures with FLS cells from acutely inflamed knee joints but without indomethacin. However, indomethacin did not prevent upregulation of NK1 receptor-like IR in co-cultures with FLS cells from chronically inflamed knee joints, consistent with the finding that the supernatant from FLS cells from chronically inflamed knee joints did not induce upregulation of NK1 receptor-like IR. By contrast, neither the addition of an antibody to IL-6 (1 μmol/l) nor addition of normal rat IgG (1 μmol/l) prevented the upregulation of NK1 receptor-like IR in the presence of FLS cells from acutely and chronically inflamed knee joints (Figure 7, each column shows the data from 4 co-cultures). These data suggest that a COX product plays an important role in the upregulation of NK1 receptor-like IR in the acute stage of inflammation but not in the chronic one.

## Discussion

The present study shows that FLS cells from the knee joint influence the expression of pain-related receptors in DRG neurones. The expression of NK1 receptors was affected only by co-culture with FLS cells from inflamed knee joints. By contrast, B2 receptors were upregulated by FLS cells from normal knee joints, and this effect was more pronounced in the co-culture with FLS cells from acutely inflamed joints. The expression of TRPV1 receptors was slightly upregulated by FLS cells from normal joints, but a significant upregulation was found only in the presence of FLS cells from inflamed joints, with the strongest effects after co-culture with FLS cells from chronically inflamed joints. The upregulation of NK1 receptor-like IR by FLS cells from acutely inflamed joints was mimicked by the supernatant of FLS cells from acutely inflamed joints, but the expression of neither B2 nor TRPV1 receptors was influenced by supernatants from FLS cells from normal, acutely or chronically inflamed knee joints. Although FLS cells from both acutely and chronically inflamed joints produced elevated levels of PGE_2_, only the upregulation of NK1 receptor-like IR by FLS cells from acutely inflamed joints was prevented by indomethacin, suggesting that prostaglandins and/or other COX products are relevant mediators for receptor regulation in acute arthritis but not in chronic arthritis.

Initially, it was important to establish optimal conditions for co-culturing FLS cells and DRG neurones. We chose a DMEM medium that is routinely used for FLS cells. However, we did omit FCS in order to avoid uncontrollable concentrations of additives, such as growth factors, and added nerve growth factor to support the development of neurites. Although the major FLS cell type was morphologically different from DRG neurones, some cells could not be identified. We therefore used an antibody to NSE in the mono-cultures and the co-cultures and found that this antibody reliably labels neurones but does not label FLS cells. According to the literature, the anti-NSE antibody labels about 95% of the neurones [48]. We therefore used this antibody for double-labelling in all experiments.

Several parameters indicate that the co-culture conditions are suitable for the survival of neurones. First, neuronal perikarya in the co-culture had a similar size distribution as perikarya in DRG mono-cultures. Second, labelling for neuronal receptors yielded similar values as for neurones in DRG mono-cultures. In the absence of an inflammatory stimulus, about 10% of DRG neurones show NK1 receptor-like IR in the co-culture system as well as DRG mono-cultures, and about 40% of the DRG neurones exhibit BK-gold binding in the co-culture system and DRG mono-cultures. Only the expression of the TRPV1 receptor was less widespread than expected. From immunohistological staining of DRG sections one would expect between 30% and 40% of the neurones to be immunopositive [49,50] but only 21% of the DRG neurones were immunopositive in mono-culture, and 28% after co-culture with FLS cells from normal knee joints. This may be due to a decrease in receptor expression over time. In fact, in DRG mono-cultures of 1 day, between 30% and 40% of the neurones show an increase in [Ca^2+^]_i _after bath application of capsaicin, an agonist of the TRPV1 receptor, indicating a higher proportion of TRPV1 receptor-positive neurones (unpublished observations), and this is in line with the literature [51,52].

A major finding of this study is that receptor expression in DRG neurones in the co-culture system was influenced similarly to that under conditions of *in vivo *inflammation. In lumbar DRGs from rats with acute AIA in the knee joint, up to 50% of the DRG neurones showed NK1 receptor expression, and about 80% of the DRG neurones showed BK-gold binding [19]. Thus, receptor upregulation in the co-culture system reached almost the same level as in the course of AIA *in vivo*. In the case of the TRPV1 receptor, the proportion of positive neurones increased by about 10% in the positive studies (see Introduction), and TRPV1 receptor upregulation in the present co-culture system was in the same range. These data show that the co-culture system of FLS cells and DRG neurones is a powerful tool for the study of interactions between inflammatory processes and primary afferent neurones.

The present data provide the important novel finding that most of the changes in the receptor expression occur only when FLS cells are present, whereas the supernatant from FLS cells was not sufficient to induce changes in receptor expression, except for the upregulation of NK1 receptor expression (see below). This finding matches our previous observation that only the expression of the NK1 receptor could be manipulated by adding inflammatory compounds to the culture medium [46] whereas the expression of BK receptors was never changed by adding inflammatory compounds (unpublished observations). The data showing receptor regulation only in the presence of non-neuronal cells such as FLS cells could be a milestone in the study of mechanisms that induce changes in neurones in the course of inflammation. The most likely explanation for the positive influence of FLS cells on neurones is that FLS cells provide signals to neurones that are absent in the supernatant. Either direct cellular contacts are required to mediate the effect of FLS cells on neurones, or FLS cells create a local milieu that is not maintained in the supernatant, or both mechanisms may be at work.

Notably, FLS cells from normal, acutely and chronically inflamed joints had different effects on receptor expression in DRG neurones. FLS cells from normal knee joints induced a small upregulation of BK and TRPV1 receptors, suggesting that the basal expression of these receptors is partly dependent on extraneuronal factors. It is possible that explant cultures of FLS cells from normal knee joints do not provide an entirely physiological milieu and, therefore, it is difficult to make firm conclusions about whether FLS cells exert trophic influences on neurones under non-inflammatory conditions. However, comparison of the effects of FLS cells from normal and inflamed joints shows the potential of FLS cells to influence neurones under inflammatory conditions. The finding that BK receptors were mainly upregulated by FLS cells from acutely inflamed joints, whereas TRPV1 receptors were mainly increased by FLS cells from chronically inflamed joints, shows the potential of this approach to define mechanisms involved in neuronal receptor regulation at different stages of inflammation.

While the expression of BK and TRPV1 receptors was entirely dependent on the presence of FLS cells, the expression of NK1 receptors was also influenced by the supernatant of FLS cells from acutely inflamed knee joints, suggesting that soluble factors play an important role. There is some evidence that prostaglandins are involved. First, PGE_2 _production is increased in FLS cells from inflamed knee joints (see also [38,39]). Second, addition of PGE_2 _to the culture medium of DRG neurones enhances the proportion of neurones with NK1 receptor-like IR through activation of adenylate cyclase and protein kinase A [43]. Third, the effect of FLS cells from acutely inflamed joints on NK1 receptor expression in DRG neurones was blocked by application of the COX inhibitor indomethacin to the co-culture. Together, these data indicate a crucial role of prostaglandins in NK1 receptor upregulation at the acute stage of inflammation.

However, this explanation is not applicable to the effect of FLS cells from chronically inflamed knee joints on NK1 receptor expression. Unlike the presence of FLS cells from chronically inflamed joints, the supernatant of FLS cells from chronically inflamed joints did not cause NK1 receptor upregulation, nor was upregulation reduced by indomethacin. These data suggest that cellular interactions are important for this effect. It is unclear why the supernatant of FLS cells from chronically inflamed joints did not induce NK1 receptor upregulation. One possibility could be that FLS cells from chronically inflamed joints secrete, in addition to PGE_2_, other mediators that counteract the PGE_2 _effect. For example, the PGE_2 _effect on NK1 receptor expression can be blocked by somatostatin, which inhibits adenylate cyclase [43].

## Conclusion

The present study addresses the regulation of receptors in DRG neurones that are involved in the generation of inflammatory pain and hyperalgesia. Substance P sensitises joint afferents for mechanical stimuli, thus inducing mechanical hyperalgesia [24,25]. BK activates and sensitizes primary afferents for mechanical and chemical stimuli [13,22,23,26] and is, therefore, an important pain mediator. The TRPV1 receptor is an ion channel that is involved in thermal inflammatory hyperalgesia [27-29]. All of these receptors are upregulated at some stage of inflammation. In the present study, we have established for the first time a co-culture system of FLS cells and DRG neurones that enables the investigation of mechanisms of interaction between cells contributing to joint pathology and neurones involved in pain and neurogenic mechanisms of inflammation. We provide evidence that three different pain-related receptors in DRG neurones are differently regulated by FLS cells and mediators from FLS cells. While extracellular soluble mediators from FLS cells from acutely inflamed knee joints are sufficient for the upregulation of NK1 receptors, the presence and most likely direct cellular contacts between FLS cells and sensory neurones are required for the upregulation of B2 and TRPV1 receptors. Importantly, the state of activity of FLS cells is crucial for their impact on neurones and, therefore, they are likely to play a pivotal role in the generation of inflammatory pain.

## Abbreviations

AIA = antigen-induced arthritis; B2 = bradykinin 2; BK = bradykinin; BSA = bovine serum albumin; BSA-C = acetylated bovine serum albumin; COX = cyclooxygenase; DMEM = Dulbecco's modified Eagle's medium; DRG = dorsal root ganglion; FCS = fetal calf serum; FLS = fibroblast-like synovial; Ig = immunoglobulin; IL = interleukin; IR = immunoreactivity; NGF = nerve growth factor; NK1 = neurokinin 1; NSE = neurone-specific enolase; PBS = phosphate-buffered saline; PGE_2 _= prostaglandin E_2_; TRPV1 = transient receptor potential V1; TX-100 = Triton X-100.

## Competing interests

The authors declare that they have no competing interests.

## Authors' contributions

GSvB and JR produced cultures of DRG neurones and established the co-culture system. They also carried out the labelling of neurones. MH, CR and RB induced the antigen-induced arthritis and produced the cultures of FLS cells, carried out the ELISA study and collaborated in establishing the co-culture system. HGS was involved in planning the study and the preparation of the manuscript.

## Figures and Tables

**Table 1:** Concentration of IL-6 and PGE_2 _in supernatants from cultured FLS cells and co-cultures from FLS cells and DRG neurones

	PGE_2 _(ng/ml)	IL-6 (ng/ml)
DRG neurones	ND	ND
FLS cells, normal knee joints	4.8 ± 2.1	10.6 ± 2.2
FLS cells, acutely inflamed knee joints	28.6 ± 5.2	22.0 ± 4.2
FLS cells, chronically inflamed knee joints	92.9 ± 23.4	NM
DRG neurones + FLS cells, normal knee joints	11.7 ± 3.1	12.8 ± 2.0
DRG neurones + FLS cells, acutely inflamed joints	39.0 ± 4.1	26.0 ± 3.9
DRG neurones + FLS cells, chronically inflamed joints	75.7 ± 18.5	NM

DRG, dorsal root ganglion; FLS, fibroblast-like synovial; IL, interleukin; ND, not detected; NM, not measured; PGE_2_, prostaglandin E_2_.
